# 3-in-one ^PD-1^CAR Tregs: A bioengineered cellular therapy for target engagement, activation, and immunosuppression with reparative potential

**DOI:** 10.1016/j.isci.2025.113677

**Published:** 2025-10-03

**Authors:** Shuyun Dong, Tianxiao Zhang, Yujia Zhai, Lauren C. Naatz, Noel G. Carlson, John W. Rose, Brian Evavold, Mingnan Chen

**Affiliations:** 1 Department of Molecular Pharmaceutics, University of Utah, Salt Lake City, UT 84124, USA; 2 Department of Neurology, Division of Neuroimmunology and Autoimmune Neurology, University of Utah, Salt Lake City, UT 84124, USA; 3 Department of Neurology, University of Utah, Salt Lake City, UT 84124, USA; 4 GRECC, VA Salt Lake City Health Care System, Salt Lake City, UT 84124, USA; 5 Department of Pathology, University of Utah, Salt Lake City, UT 84124, USA

**Keywords:** Immunology

## Abstract

Chimeric Antigen Receptor (CAR) regulatory T cells (Tregs) represent a promising cell-based therapy for autoimmune diseases, yet conventional designs require multistep activation before suppressing pathogenic cells, limiting precision and efficacy. We developed a “3-in-One” CAR Treg platform targeting Programmed cell death protein 1 (PD-1), enabling direct engagement with, activation by, and suppression of PD-1^+^ effector conventional T cells (eTconvs), key drivers of autoimmune inflammation. ^PD-1^CAR Tregs stably expressed CAR and FoxP3, displayed high CD25, and upon PD-1 engagement, upregulated Ki67, IL-10, and TGF-β1 without producing IFNγ or IL-2, maintaining a committed regulatory phenotype. Functionally, they inhibited T cell proliferation and preferentially reduced PD-1^+^ eTconvs, a specificity not seen in conventional Tregs. Moreover, ^PD-1^CAR Tregs promoted oligodendrocyte precursor cell differentiation and secreted CCN3, a reparative factor, suggesting dual benefits in immune regulation and neuronal repair. These findings establish ^PD-1^CAR Tregs as a unique therapy with both targeted suppression and tissue repair potential.

## Highlights

• “3-in-One” ^PD-1^CAR Tregs enable direct engagement, activation, and suppression
• ^PD-1^CAR Tregs maintain FoxP3, high CD25, and secrete IL-10/TGF-β1 but not IFNγ or IL-2
• Upon PD-1 activation, ^PD-1^CAR Tregs suppress PD-1^+^ effector T cells
• ^PD-1^CAR Tregs secrete CCN3 and promote OPC differentiation to support repair

## Graphical abstract

**Figure:**
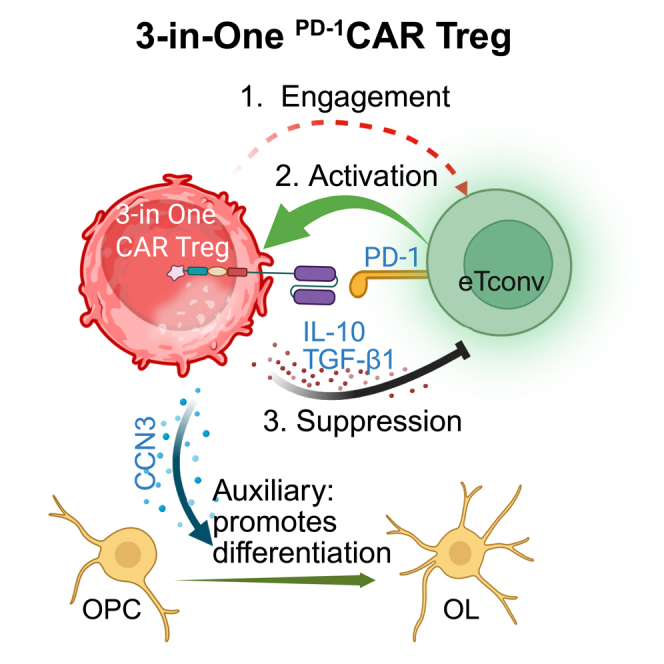

## Introduction

The immune system functions to defend the body from infections and cancer while maintaining tolerance to self. When this balance breaks down, the immune system can mistakenly attack the body’s own tissues, leading to autoimmune diseases (ADs) such as multiple sclerosis (MS), type 1 diabetes, and rheumatoid arthritis. These chronic diseases often require long-term immunosuppression, which carries risks of systemic side effects and incomplete control. As an alternative, cell-based immunotherapies have emerged to selectively reprogram or restrain overactive immune responses. One of the most promising approaches involves the use of regulatory T cells (Tregs)—a specialized subset of CD4^+^ T cells that naturally suppress immune activation and maintain immune tolerance. Tregs have emerged as a promising therapeutic strategy for establishing and restoring immunotolerance in a wide range of clinical settings, including ADs and organ transplantation.^1^^,^^2^^,^^3^^,^^4^^,^^5^ Among Treg-based strategies, engineered chimeric antigen receptor (CAR) Tregs offer notable advantages over endogenous Tregs due to their enhanced potency, customizability, and reliable sourcing.^1^^,^^5^^,^^6^ These features make CAR Tregs particularly appealing for therapeutic applications requiring targeted and sustained immune regulation.

Despite this promise, current CAR Treg therapies face important limitations in efficacy, particularly in achieving durable and localized immunotolerance.^6^ Most existing CAR Tregs are designed to recognize and be activated by soluble or membrane-bound auto-or allo-antigens associated with disease, but not expressed directly on pathogenic immune cells.^5^^,^^6^ The function of these antigen specific CAR Tregs relies on a multi-step process: antigen engagement, cellular activation, and subsequent migration—defined here as local positioning toward pathogenic effector cells—to exert their immunosuppressive effects. Because they rely on this indirect mechanism, we refer to them as indirect targeting CAR Tregs (IndiCAR Tregs). The sequential mechanism of IndiCAR Tregs introduces delays in therapeutic action, compromises spatial precision, and may limit overall clinical efficacy. These shortcomings are especially problematic in complex autoimmune conditions such as MS, where pathogenic autoreactive effector conventional T cells (eTconvs) drive chronic neuroinflammation.^7^^,^^8^^,^^9^ IndiCAR Tregs designed to potentially treat MS typically target myelin antigens such as myelin oligodendrocyte glycoprotein (MOG) or myelin basic protein (MBP).^10^^,^^11^^,^^12^^,^^13^ However, the mere presence of myelin does not reliably indicate active demyelination or the local presence of pathogenic eTconvs. As a result, IndiCAR Tregs must migrate into close proximity with eTconvs to exert their immunosuppressive effects—creating a spatial and temporal bottleneck for therapeutic action. Moreover, identifying relevant driver antigens is often impractical due to disease heterogeneity and the poorly defined origins of many autoimmune conditions.^6^^,^^14^ These challenges highlight the urgent need for a next-generation, bioengineered Treg therapy that can engage, respond to, and suppress pathogenic immune cells in a rapid and spatially relevant manner.

To address these limitations, we developed a “3-in-One” CAR Treg system targeting Programmed Death-1 (PD-1), an immune checkpoint receptor selectively upregulated on activated—but not naïve— immune cells, primarily eTconvs, as well as effector B cells and other subsets during inflammation.^15^^,^^16^^,^^17^^,^^18^^,^^19^ PD-1^+^ cells, collectively, propel immune responses across a range of conditions, including ADs, cancers, and transplant rejection.^20^^,^^21^^,^^22^^,^^23^^,^^24^^,^^25^^,^^26^^,^^27^^,^^28^^,^^29^^,^^30^^,^^31^^,^^32^ In patients with AD^23^^,^^24^^,^^26^^,^^27^ and animal models,^19^^,^^28^^,^^29^^,^^33^^,^^34^^,^^35^^,^^36^^,^^37^ PD-1^+^ eTconvs are more prevalent, infiltrate inflamed tissues, and release pathogenic cytokines. Notably, PD-1 blockade—while effective in oncology—has been shown to exacerbate autoimmunity and transplant rejection in both clinical and preclinical studies by unleashing immune activation.^20^^,^^21^^,^^28^^,^^29^^,^^30^^,^^31^ Conversely, the targeted depletion of PD-1^+^ cells eliminates pathogenic immune populations and therefore reduces disease severity of ADs.^38^^,^^39^^,^^40^ These findings establish PD-1^+^ immune cells as compelling targets for selective immunosuppression.

Our “3-in-One” PD-1 targeting CAR Treg (^PD-1^CAR Treg) design integrates target engagement, activation, and suppression into a single cell-intrinsic mechanism. In this design, autoreactive PD-1^+^ eTconvs simultaneously serve as: (1) Engagers, recruiting CAR Tregs to sites of immune activation; (2) Activators, triggering Treg suppressive programs upon binding; and (3) Targets, receiving direct immunosuppressive effects. This bioengineered approach bypasses the need for intermediate signals or tissue migration, delivering precision immunomodulation at the point of inflammation.

In addition to their immunosuppressive function, Tregs are increasingly recognized for their role in tissue repair and regeneration, including in the central nervous system (CNS).^41^^,^^42^^,^^43^^,^^44^^,^^45^^,^^46^ They can secrete reparative factors such as CCN3 and osteopontin to promote wound healing and neural recovery.^43^^,^^45^^,^^46^^,^^47^^,^^48^ In the context of MS, the remyelination of damaged axons is essential for restoring neurological function.^49^^,^^50^ Prior studies have shown that Treg-derived CCN3 supports remyelination by promoting the differentiation of oligodendrocyte precursor cells (OPCs) into mature, myelin-producing oligodendrocytes (OLs).^47^^,^^48^^,^^51^ Incorporating this regenerative capacity into engineered Tregs could provide dual therapeutic value in diseases characterized by both immune dysregulation and tissue degeneration.

In this study, we designed, produced, and validated ^PD-1^CAR Tregs using both engineered T cell lines and primary mouse T cells. We demonstrate that ^PD-1^CAR Tregs engage their PD-1^+^ target cells, become selectively activated, suppress eTconv proliferation, and avoid pro-inflammatory cytokine production. Furthermore, these cells secrete CCN3 and promote OPC differentiation into mature OLs, demonstrating a potential dual-function capacity for both immune suppression and neuroregeneration.

Together, our findings establish ^PD-1^CAR Tregs as a next-generation bioengineered cell therapy that integrates targeted immunosuppression with reparative potential. This “3-in-One” CAR Treg platform represents a modular and adaptable immunoregulatory system suitable for biomaterial-compatible delivery and application in autoimmune diseases such as MS, where both immune control and CNS repair are critical.

## Results

### Design and generation of ^PD-1^CAR Tregs

#### Design and lentivirus production

**Figure 1:**
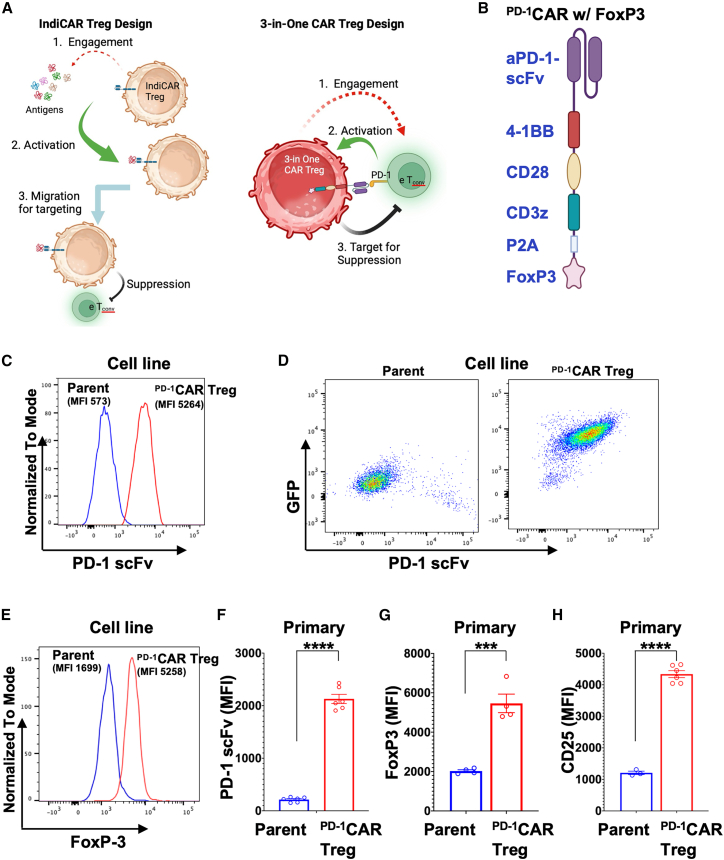
Design and generation of ^PD-1^CAR Tregs from a ^PD-1^KO mouse CD4^+^ cell line and primary CD4^+^ cells (A) Schematic illustrating the 3-in-One ^PD-1^CAR Treg design, which enables direct engagement, activation by, and immunosuppression of PD-1^+^ eTconvs, in contrast to conventional IndiCAR Tregs that require antigen recognition and activation before migrating to the vicinity of effector cells to exert their suppressive function. (B) Schematic of the ^PD-1^CAR construct, highlighting key design features. Illustrations of (A) and (B) were created using BioRender.com. (C) Representative flow cytometry histograms shows scFv expression in ^PD-1^CAR Tregs derived from PD-1^KO^ 2D2 cells, compared to parent cells. (D) Representative flow cytometry dot plots illustrates the co-expression of GFP and scFv in ^PD-1^CAR Tregs derived from ^PD-1^KO 2D2 cells, compared to parent cells. (E) Representative flow cytometry histograms show FoxP3 expression in ^PD-1^CAR Tregs derived from ^PD-1^KO 2D2 cells, compared to parent cells. (F) Bar graphs summarizing the MFI of scFv on ^PD-1^CAR Tregs derived from primary CD4^+^ cells of PD-1^KO^ mice, compared to parent cells. (G) Quantification of FoxP3 expression, presented as MFI, of ^PD-1^CAR Tregs derived from primary CD4^+^ cells from PD-1^KO^ mice, compared to parent cells. (H) Measurement of CD25 expression, presented as MFI, in ^PD-1^CAR Tregs derived from primary CD4^+^ cells from PD-1^KO^ mice, compared to parent cells. Data of (F–H) are mean ± SEM, n = 3–6. ∗∗∗*p* < 0.001, ∗∗∗∗*p* < 0.0001, as determined by Student’s two-tailed *t* test.

To address the limitations of IndiCAR Tregs, which require a multi-step process involving antigen recognition, activation, and migration before exerting suppressive function (Figure 1A, left), we developed a “3-in-1” CAR Treg strategy that enables direct, one-step engagement, activation, and suppression of pathogenic immune cells (Figure 1A, right). To test this approach, we designed PD-1-specific CAR Tregs using a single-chain variable fragment (scFv) derived from the anti-mouse PD-1 antibody (Clone 1A12). This CAR construct, expressed under the MSCV promoter, contained the intracellular domain of 4-1BB to enhance Treg persistence, along with CD28 and CD3ζ signaling domains to promote activation and proliferation (Figure 1B). To introduce and maintain the Treg lineage and functionality, a mouse FoxP3 was included downstream of the CAR, separated by a P2A self-cleaving peptide. Additionally, a GFP reporter, driven by the EF1α promoter, was integrated to enable transduction tracking and cell selection. The lentiviral plasmid carrying the CAR, along with packaging and envelope plasmids, was co-transfected into Lenti-X cells to generate ecotropic envelope-pseudotyped lentivirus for the transduction of murine cells.

#### Generation of ^PD-1^CAR Tregs from a mouse CD4^+^ cell line

To validate this construct, we first tested it in the 2D2 hybridoma cell line, which expresses CD3 and CD4 and retains antigen specificity similar to primary mouse CD4^+^ T cells. To prevent potential mutual inhibition by endogenous PD-1 expression, the PD-1 gene was knocked out using the CRISPR-Cas9 system, and the absence of PD-1 expression was confirmed via flow cytometry analysis (Figure S1). The packaged lentiviral ^PD-1^CAR was transduced into PD-1^KO^ 2D2 cells. GFP^+^ transduced cells were sorted by flow cytometry and maintained for at least 10 generations without loss of GFP expression (Figure S2A). To confirm successful CAR expression, we stained the cells for scFv using an iFluo 647-labeled anti-scFv antibody. Robust surface scFv expression was detected in GFP^+^ cells, with a mean fluorescence intensity (MFI) of 5264, but not in untransduced GFP^−^ parent cells (MFI 573) (Figure 1C). Dual staining confirmed that all GFP^+^ cells co-expressed scFv, establishing GFP as a reliable marker for CAR expression and selection (Figure 1D). Additionally, intracellular staining revealed that GFP^+^ cells expressed significantly higher FoxP3 levels (MFI 5258) compared to untransduced GFP^−^ cells (MFI 1699) (Figure 1E), confirming successful transgenic FoxP3 expression as a defining marker of the Treg phenotype.

#### Generation of ^PD-1^CAR Tregs from primary CD4^+^ cells of PD-1^KO^ mice

Although the 2D2-derived ^PD-1^CAR Tregs validated the construct design, cell lines have limitations, including potential immunogenicity and lack of natural Treg cytokine secretion. Indeed, 2D2-derived ^PD-1^CAR Tregs lacked key immunoregulatory molecules, such as CD25 expression and IL-10 secretion (data not shown), limiting their physiological relevance. To generate biologically relevant ^PD-1^CAR Tregs, we transduced primary CD4^+^ T cells isolated from PD-1^KO^ mice using the same lentiviral system. Five days post-transduction, GFP^+^ cells were sorted via flow cytometry and expanded for an additional three days before characterization or functional assays. Similar to 2D2-derived CAR Tregs, primary ^PD-1^CAR Tregs maintained stable GFP expression (Figures S2B and S2C). Surface staining confirmed robust scFv expression, with an MFI of 2129 compared to an MFI of 213 in parent cells (Figure 1F). Intracellular staining also verified FoxP3 expression as expected, with MFI of 5462 versus 2024 in the parent cells (Figure 1G). Unlike ^PD-1^CAR Tregs derived from the 2D2 cell line, which did not express CD25, a key IL-2 receptor highly expressed by Tregs, primary ^PD-1^CAR Tregs exhibited a 3.6-fold increase in CD25 expression compared to untransduced parent cells, with an MFI of 4338 vs. 1208 (Figure 1H).

Together, these results confirm the successful design, lentiviral production, and stable expression of ^PD-1^CAR and FoxP3 in the engineered Tregs, establishing a reliable platform for further functional evaluation of this bioengineered cellular therapy.

### ^PD-1^CAR Tregs recognize and specifically bind programmed cell death protein 1 for engagement

**Figure 2:**
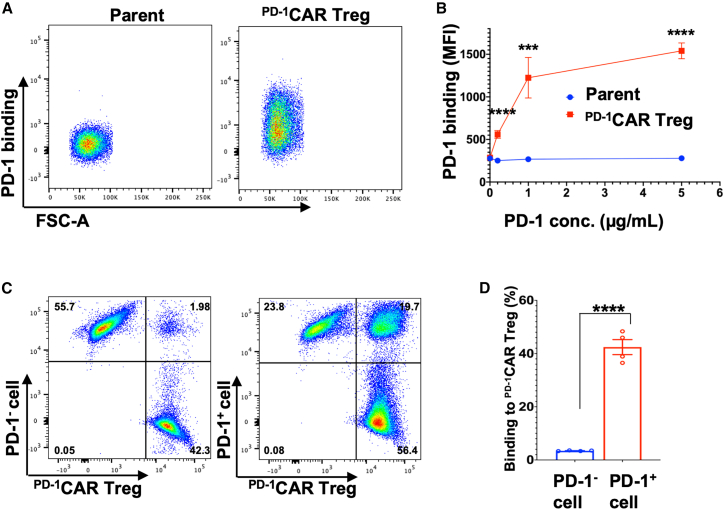
^PD-1^CAR Tregs Bind PD-1 for Engagement (A) Representative flow cytometry dot plots show PD-1 binding on 2D2 cell line-derived ^PD-1^CAR Tregs, but not on parent 2D2 PD-1^KO^ cells. (B) Dose-dependent binding of PD-1, represented as MFI, on 2D2 cell line-derived ^PD-1^CAR Tregs, with no detectable binding on parent 2D2 PD-1^KO^ cells. (C) Representative flow cytometry dot plots show double-positive (CFSE^+^/eFluor 670^+^) cell conjugates, indicating the binding of CFSE-labeled 2D2 cell line-derived ^PD-1^CAR Tregs with eFluor 670-labeled PD-1^+^ or PD-1^−^ EL4 cells. (D) Bar graph quantifying the percentage of CFSE^+^/eFluor 670^+^ double-positive cell conjugates formed between PD-1^+^ EL4 cells co-cultured with 2D2 cell line-derived ^PD-1^CAR Tregs at a ratio of 1:1, compared to conjugates formed with PD-1^−^ EL4 cells. Data of (B) and (D) are mean ± SEM, *n* = 4; ∗∗∗*p* < 0.001, ∗∗∗∗*p* < 0.000, as determined by Student’s two-tailed *t* test.

To assess whether the scFv expressed on ^PD-1^CAR is functional in recognizing and binding its target PD-1, a critical first step in the “3-in-One” strategy, we performed a cell-molecule binding assay. Either 2D2-derived ^PD-1^CAR Tregs or control parent cells were incubated with His-tagged PD-1 protein, allowing for potential binding to the cell surface. After incubation, the cells were washed, stained with an APC-*anti*-His tag antibody, and analyzed by flow cytometry to determine which cells were associated with PD-1. The results confirmed that ^PD-1^CAR Tregs specifically bound to PD-1, whereas parent cells did not (Figure 2A). Binding to PD-1 was dose-dependent, with ^PD-1^CAR Tregs showing an MFI of 560 at 0.2 μg/mL, compared to a baseline MFI of 279 without PD-1 treatment. Increasing the PD-1 concentration to 1 μg/mL and 5 μg/mL further elevated the MFI to 1225 and 1540, respectively. In contrast, parent cells showed minimal binding at all concentrations, with MFI values consistently below 280 (Figure 2B).

Further, to directly assess cell-cell interactions, we performed a cell conjugation assay. CFSE-labeled ^PD-1^CAR Tregs were co-cultured with eFluor 670-labeled EL4 cells that highly express PD-1, or EL4 PD-1^ko^ cells as a control. After co-incubation, paraformaldehyde (PFA) was added to fix potential cell-cell interactions. Flow cytometry analysis quantified double-positive (CSFE^+^/eFluor 670^+^) cell conjugates, representing ^PD-1^CAR Tregs bound to PD-1^+^ target cells. As shown in Figure 2C, a significant number of cell conjugates were observed in the co-culture with PD-1^+^ EL4 cells, while only baseline levels of conjugates were detected with ^PD-1^KO EL4 cells. Quantification revealed that at 1:1 ratio co-culture, approximately 42.43% of PD-1^+^ EL4 cells bound to PD-1^+^ cells, whereas only 3.44% of binding cells were observed in the PD-1^KO^ EL4 (Figure 2D).

These data demonstrate that the PD-1 scFv is functionally expressed on the surface of CAR Tregs, allowing them to specifically recognize and engage their target, the PD-1 molecule, and bind to PD-1^+^ cells. This functionality provides a critical foundation for the “3-in-One” strategy, enabling ^PD-1^CAR Tregs to directly interact with PD-1^+^ cells, thereby facilitating subsequent activation and immunosuppressive function.

### ^PD**-**1^CAR Tregs are activated by programmed cell death protein 1^+^ target cells and secrete IL-10 and TGF-β1, but not IFN-γ and IL-2

**Figure 3:**
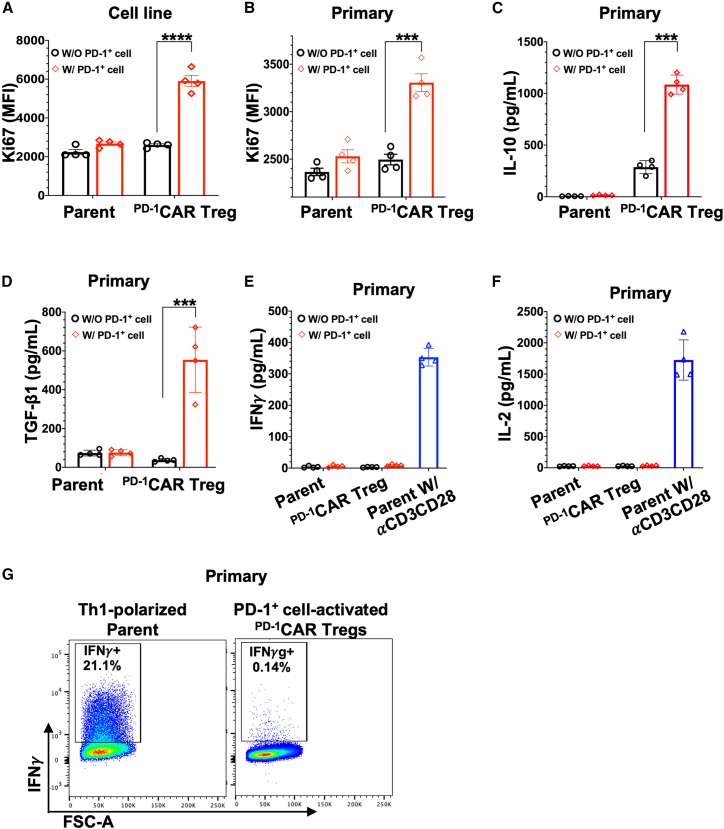
^PD**-**1^CAR Tregs are activated by PD-1^+^ target cells and secrete IL-10 and TGF-β1, but not IFN-γ and IL-2 (A) Bar graph summarizing Ki67 expression in 2D2 ^PD-1^KO cells and 2D2 cell line-derived ^PD-1^CAR Tregs with and without the stimulation of PD-1^+^ cells at a 1:1 ratio for three days. (B) Bar graph summarizing Ki67 expression in primary parent cells and ^PD-1^CAR Tregs with and without stimulation PD-1^+^ cells at a 1:1 ratio for three days. (C–E and G) Bar graph summarizes cytokine secretion by primary parent cells and ^PD-1^CAR Tregs with and without PD-1^+^ cell stimulation (1:1) for three days. (C) IL-10, (D) TGF-β1, (E) IFN-γ, and (G) IL-2. (F) Flow cytometry dot plots show IFN-γ expression in primary ^PD-1^CAR Tregs activated by PD-1^+^ cells at a 1:1 ratio for three days, compared to parent cells under Th1 polarization conditions as a control. Data of (A–E and G) are mean ± SEM, *n* = 4; ∗∗∗*p* < 0.001, ∗∗∗∗*p* < 0.0001, as determined by Student’s two-tailed *t* test.

To assess the activation step of our “3-in-One” strategy, we examined whether ^PD-1^CAR Tregs could be directly activated by PD-1^+^ target cells. PD-1^+^ target cells were generated by stimulating freshly isolated CD4^+^ T cells from 2D2 transgenic mouse with plate-bound anti-CD3/anti-CD28, resulting in robust PD-1 expression (Figure S3). These PD-1^+^ cells were then irradiated with X-ray to prevent proliferation and co-cultured with ^PD-1^CAR Tregs at a 1:1 ratio for three days. Treg activation was evaluated by measuring Ki67 expression. We evaluated both 2D2-derived and primary ^PD-1^CAR Tregs and found that each exhibited activation in response to PD-1^+^ target cells, as indicated by increased Ki67 expression. Without stimulation, Ki67 levels in 2D2-derived ^PD-1^CAR Tregs were similar to those in parent 2D2 ^PD-1^KO cells (MFI: 2601 vs. 2235, respectively). Following co-culture with PD-1^+^ cells, Ki67 MFI in ^PD-1^CAR Tregs increased markedly to 5901—a 2.27-fold increase—whereas the parent cells showed only a modest rise to 2673 (Figure 3A). Similarly, primary ^PD-1^CAR Tregs exhibited a baseline Ki67 MFI of 2495, comparable to 2365 in parent CD4^+^ cells. After co-culture with PD-1^+^ targets, Ki67 expression in primary ^PD-1^CAR Tregs increased significantly to 3306, though to a lesser extent than in 2D2-derived ^PD-1^CAR Tregs. In contrast, the parent primary CD4^+^ cells showed only a slight increase to 2530 (Figure 3B).

To further characterize the functional responses of ^PD-1^CAR Tregs, we examined cytokine secretion following co-culture with PD-1^+^ target cells. Both IL-10 and TGF-β1, key inhibitory cytokines associated with Treg function, were markedly elevated in the supernatants after co-culture. IL-10 increased from 286 pg/mL to 1078 pg/mL (a 3.77-fold increase), while total TGF-β1 rose from 36.98 pg/mL to 553.89 pg/mL (nearly a 15-fold increase). By contrast, parent CD4^+^ T cells secreted no detectable IL-10 and maintained only baseline levels of total TGF-β1 (∼80 pg/mL) regardless of co-culture (Figures 3C and 3D).

In contrast to these suppressive cytokines, both IFN-γ and IL-2 were essentially undetectable in ^PD-1^CAR Tregs and parent CD4^+^ T cells before and after co-culture, while, as positive controls, CD4^+^ T cells stimulated with anti-CD3/CD28 produced ∼350 pg/mL of IFN-γ and 1726 pg/mL of IL-2 (Figures 3E and 3F). Flow cytometry analysis further confirmed that primary ^PD-1^CAR Tregs produced almost no IFN-γ upon stimulation (0.14%), while parent CD4^+^ T cells under Th1-polarizing conditions robustly produced IFN-γ (21.1%), validating the assay (Figure 3G).

Together, these findings support the activation step of our “3-in-One” strategy, demonstrating that ^PD-1^CAR Tregs engage with and are functionally activated by PD-1^+^ target cells, leading to an enhanced immunosuppressive phenotype.

### ^PD**-**1^CAR Tregs suppress target cell proliferation and preferentially reduce the proportion of programmed cell death protein 1^+^ T cells

**Figure 4:**
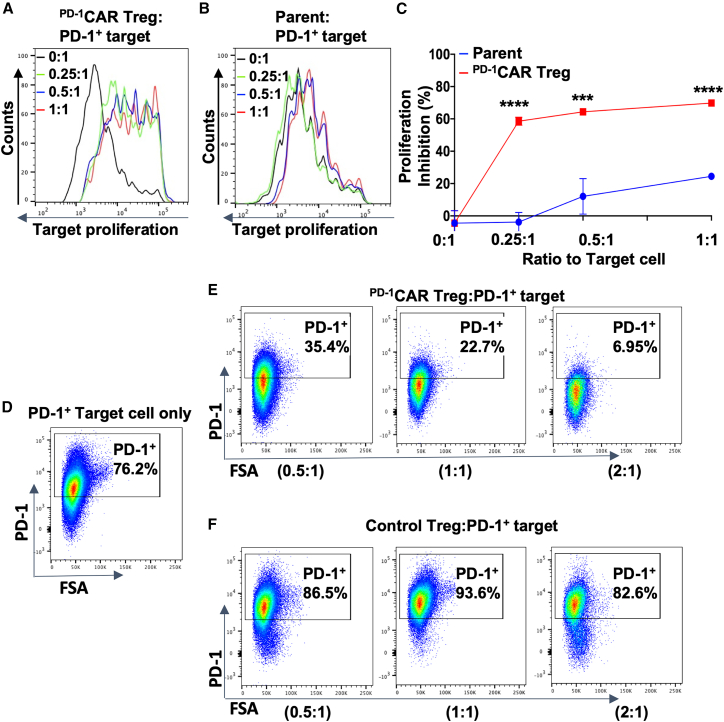
^PD**-**1^CAR Tregs inhibit primary CD4^+^ T cell proliferation and preferentially reduce the proportion of PD-1^+^ target cells (A and B) Representative flow cytometry histograms show the proliferation of mouse CD4^+^ T cells (pre-activated overnight with anti-CD3/CD28 beads at a 1:8 bead-to-cell ratio, followed by bead removal) co-cultured for 4 days with either primary ^PD-1^CAR Tregs (A) or parent cells (B) at varying ratios. (C) Quantification of proliferation inhibition based on the division index (DI) calculated from data shown in (A) and (B). (D–F) Representative flow cytometry dot plots show PD-1 expression on PD-1^+^ target cells after four days of culture: (D) target cells alone, (E) target cells co-cultured with primary ^PD-1^CAR Tregs, and (F) target cells co-cultured with control polyclonally induced Tregs. PD-1^+^ target cells of (D–F) were generated by pre-activating primary CD4^+^ T cells isolated from 2D2 transgenic mice with 3 μg/mL plate-bound anti-CD3/CD28 for three days and then labeled with CellTrace Far Red before the co-culture. Data of (C) are mean ± SEM, *n* = 3; ∗∗∗*p* < 0.001, ∗∗∗∗*p* < 0.0001, as determined by Student’s two-tailed *t* test.

To test the immunosuppressive function, the final step of our “3-In-One” strategy, we examined whether primary ^PD-1^CAR Tregs could suppress the proliferation of targeting immune cells. To prepare target cells, CD4^+^ T cells from C57BL/6 mice were labeled with CellTrace Far Red and pre-activated overnight with anti-CD3/CD28 beads. After bead removal, target cells were co-cultured with primary ^PD-1^CAR Tregs or parent control CD4^+^ T cells at varying ratios for four days. Target cell proliferation was assessed by Far Red dye dilution. ^PD-1^CAR Tregs strongly suppressed CD4^+^ T cell proliferation in a dose-dependent manner (Figure 4A), whereas parent cells showed only mild effects (Figure 4B). Quantitatively, ^PD-1^CAR Tregs reduced proliferation by 58.55%, 64.38%, and 70% at 0.25:1, 0.5:1, and 1:1 ratios, respectively, while parent cells showed only 0%, 12.05%, and 24.46% inhibition across the same ratios (Figure 4C).

To further evaluate the direct impact of ^PD-1^CAR Tregs on PD-1^+^ eTconvs, we used pre-activated CD4^+^ T cells from 2D2 transgenic mice as target cells. The target cells were stimulated with plate-bound anti-CD3/CD28 for three days to induce high PD-1 expression as done previously, then co-cultured with Tregs at varying ratios for four days. The results showed that primary ^PD-1^CAR Tregs only modestly reduced proliferation, likely due to the robust expansion of highly activated target cells (Figure S4). However, increasing the ratio of primary ^PD-1^CAR Tregs led to a marked and dose-dependent reduction in total live target cells, from 152k at a 0:1 ratio to 108k, 49k, and 23k at 0.5:1, 1:1, and 2:1, respectively (Table 1). Importantly, the percentage of PD-1^+^ cells within the surviving target population declined significantly, from 76.2% at a 0:1 ratio to 35.4%, 22.7%, and 6.95% at 0.5:1, 1:1, and 2:1, respectively (Figures 4D and 4E). In contrast, conventional Tregs without CAR expression did not exhibit PD-1^+^-specific inhibition. Although total live target cell numbers decreased—from 152k to 107k, 81k, and 41k (Table 1), the proportion of PD-1^+^ cells remained consistently high (>80%) at all ratios (Figure 4F). Further study showed that co-culture with activated ^PD-1^CAR Tregs induced more apoptosis in PD-1^+^ target cells than unactivated CAR Tregs or parent control cells (Figure S5), suggesting that part of the reduction in PD-1^+^ target cells may be due to increased cell death.

**Table 1:** Survived total target cell numbers after coculture with Tregs

Ratio to PD-1^+^ target	Control Treg	^PD-1^CAR Treg
0:1	152,331 ± 17,921	152,331 ± 17,921
0.5:1	107,164 ± 2,476	107,864 ± 3,865
1:1	81,162 ± 2,340	48,904 ± 4,075
2:1	40,685 ± 1,402	23,122 ± 2,391

Together, these findings demonstrate that ^PD-1^CAR Tregs suppress the proliferation of immune T cells and selectively target and decrease proportion PD-1^+^ target cells in a dose-dependent manner. This supports the “immune suppression step” of our “3-In-One” strategy, highlighting the unique capacity of ^PD-1^CAR Tregs to modulate pathogenic immune responses.

### ^PD**-**1^CAR Tregs promote oligodendrocyte precursor cell differentiation into oligodendrocytes for remyelination and secrete repairing factor CCN3

**Figure 5:**
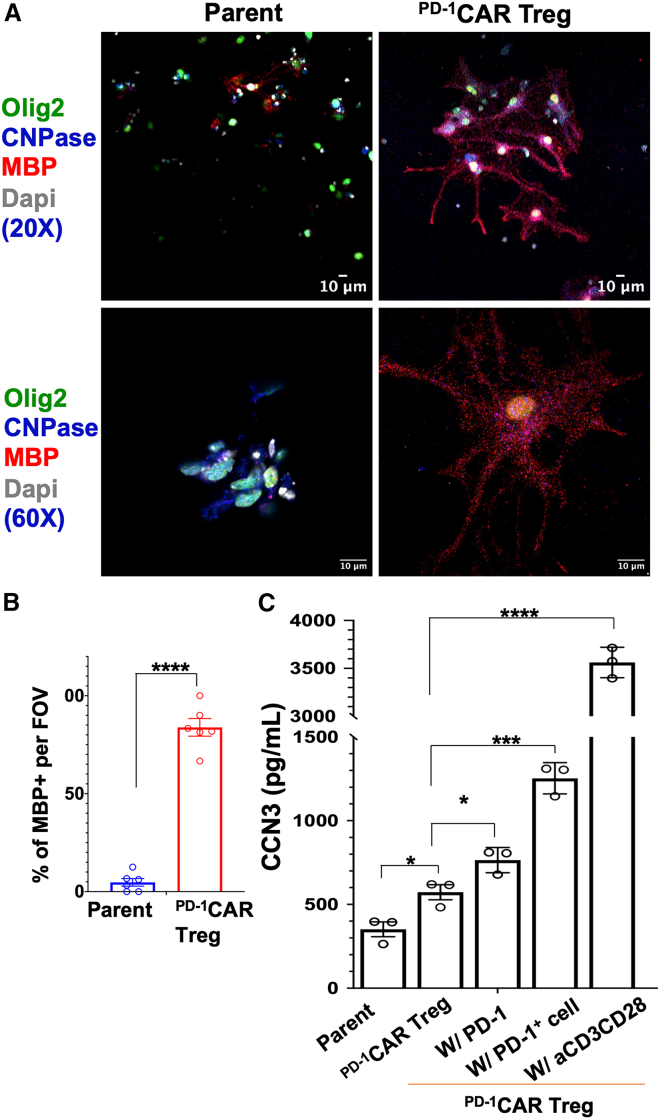
^PD**-**1^CAR Tregs promote the differentiation of OPCs into OLs and enhance release of CCN3 (A) Representative images of OPC cultures treated with activated ^PD-1^CAR Treg- or control parent cell-conditioned media for 3 days, captured at 20X and 60× magnification. Green: Olig2, Blue: CNPase, Red: MBP. Scale bars: 10 μm. Data are representative of at least six independent samples. (B) Bar graphs summarize the quantification of the percentage of MBP^+^ cells in total Olig2^+^ cells per field of view (FOV, 0.1 mm^2^). (C) Bar graphs summarize the ELISA quantification of CCN3 secreted by control cells (parent primary CD4^+^ T cells) or ^PD-1^CAR Tregs with different stimulations. Data are mean ± SEM, *n* = 3. ∗*p* < 0.05, ∗∗∗*p* < 0.001, ∗∗∗∗*p* < 0.0001. Differences were analyzed using a one-way ANOVA.

Tregs have been shown to promote myelin regeneration in the CNS, a process critical for recovery in autoimmune diseases such as MS.^41^^,^^43^^,^^44^ Following the validation of the “3-in-One” function of ^PD-1^CAR Tregs, we assessed their potential role in stimulating remyelination by testing their ability to promote the differentiation of OPCs into mature OLs—a key step in myelin repair. OPCs were isolated from the cortices of neonatal B6 mice (postnatal days 7–9), cultured for seven days to proliferate, and then treated with conditioned media from either primary ^PD-1^CAR Tregs activated with PD-1^+^ cells or parent cells for an additional three days. Cells were fixed and stained for Olig2 (a transcription factor marking OPCs), CNPase (an early marker of OPC-to-OL differentiation), and MBP (a myelin protein produced by mature OLs), and analyzed via confocal microscopy. As expected, Olig2 localized primarily to the nucleus, CNPase was detected in the cell body, and MBP was observed in the dendritic processes. OPCs exposed to ^PD-1^CAR Treg-conditioned media showed enhanced morphological features of differentiation, including enlarged cell bodies, extended dendrites, and co-expression of Olig2, CNPase, and MBP. In contrast, OPCs treated with control media from parent cells remained smaller, lacked dendritic extension, and did not express MBP (Figure 5A). Quantification revealed that 83.84% of cells treated with ^PD-1^CAR Treg-conditioned media were MBP^+^, compared to only 4.76% in the control treatment (Figure 5B). These findings indicate that ^PD-1^CAR Tregs robustly promote OPC differentiation into mature, myelin-producing OLs, whereas control cells do not.

Given prior reports implicating CCN3 as a key Treg-derived factor promoting OPC differentiation,^43^^,^^47^^,^^52^ we next examined CCN3 secretion by ^PD-1^CAR Tregs. 2D2-derived ^PD-1^CAR Tregs and their parent cells did not produce detectable CCN3 (data not shown). However, primary ^PD-1^CAR Tregs secreted significantly higher levels of CCN3 compared to control parent cells. At baseline, ^PD-1^CAR Tregs secreted 572.61 pg/mL of CCN3, compared to 351.46 pg/mL by parent cells. Co-culture with PD-1^+^ target cells further increased CCN3 secretion to 1253.15 pg/mL, while stimulation with soluble PD-1 induced 764.82 pg/mL (Figure 5C). As expected, anti-CD3/CD28 stimulation elicited the highest CCN3 production at 3561.69 pg/mL, serving as a positive control (Figure 5C).

Together, these findings suggest that ^PD-1^CAR Tregs possess auxiliary pro-repair capabilities in addition to their core immunosuppressive function. Their ability to both promote OPC differentiation and secrete CCN3 supports their potential as a dual-function therapeutic in diseases such as MS, where both immune regulation and remyelination are essential for disease control and recovery.

## Discussion

This study presents a bioengineered “3-in-One” ^PD-1^CAR Treg platform that addresses limitations of conventional IndiCAR Treg therapies in autoimmune diseases. Traditional IndiCAR Tregs rely on a multi-step cascade—antigen engagement, activation, and migration to the vicinity of eTconvs—to exert immunosuppressive effects. This sequential process reduces spatial precision, delays therapeutic onset, and often limits efficacy in complex autoimmune settings. Our “3-in-One” strategy integrates target recognition, activation, and suppression into a single, cell-intrinsic process. This enables engineered Tregs to respond immediately and selectively at the site of inflammation, providing faster and more localized immune modulation. In addition to their immunoregulatory function, ^PD-1^CAR Tregs also exhibited auxiliary reparative properties, promoting OPC differentiation and secreting CCN3, thereby expanding their potential application to diseases such as MS, which require both immune suppression and tissue repair.

The “3-in-One” strategy was realized by engineering a lentiviral construct encoding a PD-1-specific CAR incorporating CD28, 4-1BB, and CD3ζ signaling domains, co-expressed with FoxP3 to convert conventional T cells into PD-1 targeting CAR Tregs.^53^^,^^54^^,^^55^ While some studies have reported that 4-1BB stimulation alone may reduce CAR Treg suppressive function due to tonic signaling, and that transient exposure to mTOR inhibitors can mitigate this effect.^56^^,^^57^^,^^58^ Other studies support the inclusion of 4-1BB—particularly in combination with CD28—to enhance CAR Treg function and stability.^53^^,^^59^ In our preliminary studies, *in vitro* comparisons between CD28-only and CD28/4-1BB CAR constructs revealed no significant differences in suppressive function, Foxp3 expression, or cytokine profile. Therefore, we focused on the dual-domain construct in this article. Ongoing *in vivo* studies are assessing potential differences in persistence and efficacy, which will be reported separately.

For the rationale for selecting PD-1 as a target to implement the “3-in-One” strategy, targeting PD-1^+^ cells offers several compelling advantages. First, because PD-1 is broadly expressed on both activated T and B cells, its targeting enables the suppression of multiple arms of the immune response.^60^ Second, since PD-1 is absent on naive immune cells, selective targeting spares the naive lymphocyte pool, preserving immune repertoire diversity and reducing the risk of long-term immunodeficiency—a major limitation of traditional immunosuppressants such as teplizumab.^61^^,^^62^ These attributes make PD-1 not only a strategic marker for targeting pathogenic eTconvs but also a valuable model for testing and demonstrating the effectiveness of our “3-in-One” CAR Treg platform.

Regarding the translational feasibility of PD-1-targeting CAR Treg using PD-1-deficient cells, the use of PD-1^KO^ in adoptive cell therapies has already shown translational promise. A PD-1^KO^ anti-CD19 CAR T cell therapy has been evaluated in a Phase I clinical trial for relapsed/refractory B cell non-Hodgkin lymphoma, demonstrating improved preclinical survival and extended *in vivo* efficacy compared to PD-1–intact CAR T cells, thereby supporting the clinical feasibility of PD-1^KO^ in engineered cell products.^63^ While clinical data for PD-1^KO^ CAR Tregs are not yet available, preclinical studies have shown that these cells maintain Foxp3 expression and suppressive function for at least three weeks post-transfer in a xenogeneic graft-versus-host disease (xenoGVHD) model.^64^ Together, these findings support the viability and translational potential of PD-1–deficient CAR Tregs for autoimmune disease therapy.

For validation, the designed CAR construct was tested in PD-1^KO^ 2D2 cell line-derived and primary CD4^+^ T cells. The 2D2 model provided a consistent and tractable platform to confirm CAR and FoxP3 expression (Figures 1C–1E) and assess target-binding specificity; indeed, PD-1 binding assays showed specific and dose-dependent engagement exclusively in 2D2-derived ^PD-1^CAR Tregs (Figure 2), confirming the first step of the “3-in-One” mechanism. In parallel, primary ^PD-1^CAR Tregs exhibited a physiologically relevant regulatory profile, including the stable expression of FoxP3 and CD25 and secretion of IL-10 and TGFβ1 upon activation, while lacking IFN-γ and IL-2 production (Figures 1F–1H and 3C–3G). Although additional cytokines remain to be examined, the current data support their suitability as a regulatory platform. Upon engaging PD-1^+^ target cells, ^PD-1^CAR Tregs demonstrated direct activation, as evidenced by Ki67 upregulation—fulfilling the second mechanistic step of the platform (Figures 3A and 3B). For the third step, suppression, co-culture assays demonstrated that ^PD-1^CAR Tregs inhibited the proliferation of primary CD4^+^ T cells in a dose-dependent manner, outperforming untransduced controls (Figures 4A–4C). Importantly, ^PD-1^CAR Tregs also selectively reduced the proportion of PD-1^+^ target cells, a feature not observed with conventional Tregs, which broadly suppress without preferential targeting (Figures 4D–4F). This selective reduction of PD-1^+^ target cells enhances the precision of ^PD-1^CAR Tregs, offering a strategic advantage in autoimmune conditions where PD-1^+^ eTconvs are key contributors to disease pathology.

We found that the function of ^PD-1^CAR Tregs is context-dependent. Their suppressive activity was assessed under two distinct target cell conditions: (1) CD4^+^ T cells mildly activated overnight with anti-CD3/CD28 beads, which contained a mixture of PD-1^+^ and PD-1^-^ cells prior to the proliferation assay, and (2) CD4^+^ T cells stimulated with plate-bound anti-CD3/CD28 for three days to induce high PD-1 expression before the assay. In the first setting, ^PD-1^CAR Tregs robustly inhibited overall T cell proliferation (Figures 4A–4C). In the second setting, where target cells were almost entirely PD-1^+^ and had already undergone strong expansion, proliferation inhibition was modest (Figure S4). Nonetheless, the total number of viable target cells was significantly reduced (Table 1), and the frequency of PD-1^+^ cells within the surviving population markedly declined, leaving most residual cells PD-1^-^ (Figures 4D–4F). Increased apoptosis of target cells was also observed in co-culture with activated ^PD-1^CAR Tregs under this condition (Figure S5). These findings suggest that ^PD-1^CAR Tregs can mediate both suppressive and possibly cytotoxic-like effects in a target-dependent manner. Treg- or CAR-mediated cytotoxicity through perforin and granzyme release has been reported previously and is not surprising to us.^65^^,^^66^ Such dual functionality may be advantageous in autoimmune disease, where selective suppression or depletion of PD-1^+^ effector cells could enhance therapeutic efficacy while maintaining overall immune homeostasis.

Beyond immunosuppression, ^PD-1^CAR Tregs displayed auxiliary reparative functions. Conditioned media from primary ^PD-1^CAR Tregs promoted OPC differentiation into mature oligodendrocytes, marked by the increased expression of MBP (Figures 5A and 5B). Simultaneously, these Tregs secreted elevated levels of CCN3, a factor associated with Treg-mediated CNS repair (Figure 5C). Although the direct causal relationship between ^PD-1^CAR Tregs released CCN3 and OPC differentiation remains to be confirmed, previous studies have shown that Tregs produce CCN3, which promotes oligodendrocyte differentiation and myelination.^43^ Notably, anti-CCN3 antibodies were shown to block Treg-induced oligodendrocyte differentiation, and CCN3-depleted Treg-conditioned media failed to promote differentiation, whereas restored CCN3 rescued this effect.^43^ The mechanistic investigation between ^PD-1^CAR Treg and secretion of CCN3 will be addressed in our follow-up study.

In conclusion, our findings establish ^PD-1^CAR Tregs as a dual-function, bioengineered therapy that combines targeted immune suppression with regenerative support. The “3-in-One” strategy redefines how CAR Tregs interface with immune pathology, offering a next-generation approach for autoimmune and neurodegenerative diseases requiring both precision and multifunctionality.

### Limitations of the study

While our study demonstrates that ^PD-1^CAR Tregs can simultaneously engage, activate, and suppress pathogenic PD-1^+^ immune cells *in vitro*, *in vivo* validation in autoimmune disease models—such as EAE for MS—is necessary to fully assess the safety, efficacy, biodistribution, and stability of these engineered cells. Additionally, although PD-1 is enriched on pathogenic effector T cells, it is also expressed on certain subsets of regulatory or exhausted immune cells; the potential off-target effects on these populations remain to be examined. A direct comparison between Foxp3-expressing CD4^+^ conventional T cells and endogenous Foxp3^+^ Tregs transduced with the same CAR-Foxp3 construct could also provide deeper mechanistic insights and should be considered in future studies. Finally, while we propose ^PD-1^CAR Tregs as a “direct” targeting strategy, a formal comparison with conventional “Indirect” CAR Tregs (e.g., MOG- or MBP-specific CARs that recognize tissue antigens rather than immune cells) is needed to clarify the relative advantages, context-specific applications, and limitations of each approach.

## Resource availability

### Lead contact

• Requests for further information and resources should be directed to and will be fulfilled by the lead contact, Shuyun Dong (shuyun.dong@utah.edu).

### Materials availability

• All unique/stable reagents generated in this study are available from the lead contact with a completed materials transfer agreement.

### Data and code availability

• All data reported in this article will be shared by the lead contact upon reasonable request.
• This article does not report original code.
• Any additional information required to reanalyze the data reported in this article is available from the lead contact upon request.

## Acknowledgments

The work was supported by the 10.13039/100000002National Institutes of Health AI139535 and AI188073 grant to Mingnan Chen (USA), and the Research Incentive Seed Grant of the College of Pharmacy, 10.13039/100007747University of Utah (10076915) to Shuyun Dong (USA). We recognize the Core Facility of University of Utah for the service of flow cytometry and cell imaging.

## Author contributions

S.D.: writing – original draft, writing – review and editing, conceptualization, methodology, investigation, and formal analysis. T.Z.: writing – review & editing, construct design, and protein structure prediction. Y.Z: writing – review and editing, animal and cell preparation. L.Z.: writing – review and editing and investigation. N.C.: writing – review and editing, and image analysis. J.W.: writing – review and editing and clinical expertise. B.E.: writing – review and editing and conceptualization. M.C.: writing – review and editing, supervision, investigation, funding acquisition, and formal analysis.

## Declaration of interests

The authors declare no competing interests.

## Declaration of generative AI and AI-assisted technologies in the writing process

During the preparation of this work, the authors used ChatGPT to check grammar and improve language. After using this tool, the authors reviewed and edited the content as needed and take full responsibility for the content of the publication.

## STAR★Methods

### Key resources table

**Table:** 

REAGENT or RESOURCE	SOURCE	IDENTIFIER
**Antibodies**		

APC anti-mouse CD279 (PD-1) Antibody	Biolegend	Cat#135209; RRID: AB_ 2251944
PE anti-mouse CD279 (PD-1) Antibody	Biolegend	Cat#135205; RRID: AB_1877232
PE/Cyanine7 anti-mouse CD25 Antibody	Biolegend	Cat#101915; RRID: AB_2616761
PE anti-mouse Ki-67 Antibody	Biolegend	Cat#652404; RRID: AB_2561525
Brilliant Violet 510™ anti-mouse CD4 Antibody	Biolegend	Cat#200553; RRID: AB_2561388
eFluor 450 anti-mouse FoxP3 (FJK-16s)	eBioscience	Cat#48-5773-82; RRID: AB_1518812
PerCP-Cyanine5.5 anti-mouse FoxP3 (FJK-16s)	eBioscience	Cat#45-5773-82; RRID: AB_914351
MonoRab™ Rabbit Anti-scFv Cocktail [iFluor 647]	GenScript	Cat#A02288
Brilliant Violet 510™ anti-mouse IFN-γ Antibody	Biolegend	Cat#505842; RRID: AB_2734494
Goat-anti-Human/Mouse/Rat Olig2 Antibody	rndsystems	Cat#AF2418-SP
APC anti-His Tag Antibody	Biolegend	Cat#362605; RRID: AB_2715818
Mouse-anti-Human/Mouse/Rat MBP Antibody	rndsystems	Cat#MAB42282
Rabbit-anti- Human/Mouse/Rat CNPase (JF10-25)	Invitrogen	Cat#MA5-32525, AB_2809802
Fluorescein (FITC) AffiniPure™ Donkey Anti-Goat IgG (H+L)	Jackson Immunoresearch	Code: 705-095-147; RRID: AB_2340401
Cy™3 AffiniPure™ Donkey Anti-Rabbit IgG (H+L)	Jackson Immunoresearch	Code: 711-165-152; RRID: AB_2307443
Cy™5 AffiniPure™ Donkey Anti-Mouse IgG (H+L)	Jackson Immunoresearch	Code: 715-175-150; RRID: AB_2340819
	This paper	N/A

**Bacterial and virus strains**		

DH5a Chemically Competent Cell	New England Biolab	Cat#C2987H

**Chemicals, peptides, and recombinant proteins**		

Recombinant human IL-2	Goldbio	Cat#1110-02-100
Recombinant Mouse PD-1 His-tag Protein	R&D systems	Cat# 9047-PD-100
Dynabeads™ Mouse T-Activator CD3/CD28 for T-Cell Expansion and Activation	Thermo Fisher	Cat#11456D
Zombie Green™ Fixable Viability Kit	Biolegend	Cat#423111
Zombie Violet™ Fixable Viability Kit	Biolegend	Cat#423113
DAPI	invitrogen	Cat#D21490
PE Annexin V	Biolegend	Cat#640908
Cell Proliferation Dye eFluor™ 670	eBioscience	Cat#65-0840-85
CFSE	eBioscience	Cat#65-0850-84
Brefeldin A Solution (1,000X)	Biolegend	Cat#420601
Ionomycin	Stemcell Technologies	Cat#73722
Fetal bovine serum (FBS)	Sigma-Aldrich	Cat#F9423
Tripsin-EDTA (0.25%)	Thermo Fisher	Cat#25200056
L-Glutamine (200mM)	Gibco	Cat#25030081
RPMI 1640 medium	Gibco	Cat#11875093
DMEM/high glucose medium	Gibco	Cat#C11995500BT
Poly-D-lysine hydrobromide	Sigma-Aldrich	Cat#P0899
Poly-L-lysine hydrobromide	Sigma-Aldrich	Cat#P1274
MACS® Neuro Medium	Miltenyi biotec	Cat#130-093-570
MACS® NeuroBrew®-21	Miltenyi biotec	Cat#130-093-566
Human PDGF-AA	PeproTech	Cat#100-13A-10UG
Human FGF-basic (FGF-2/bFGF) (154 aa)	PeproTech	Cat#100-18B-50UG
Paraformaldehyde	Sigma-Aldrich	Cat#P6148

**Critical commercial assays**		

Neural Tissue Dissociation Kits	Miltenyi biotec	Cat#130-092-628
CD140a (PDGFRα) MicroBead Kit, mouse	Miltenyi biotec	Cat#130-101547
CD4^+^ T Cell Isolation Kit, mouse	Miltenyi biotec	Cat#130-104-454
CellTrace™ Far Red Cell Proliferation Kit, for flow cytometry	Invitrogen	Cat#C34572
Mouse IL-10 DuoSet ELISA	rndsystems	Cat#DY417-05
Mouse TGF-beta 1 DuoSet ELISA	rndsystems	Cat#DY1679-05
Mouse NOV/CCN3 DuoSet ELISA	rndsystems	Cat#DY1976
LEGENDplex™ Multi-Analyte Flow Assay Kit	Biolegend	Cat#740819
Lenti-X GoStix Plus	TakaRa	Cat#631280
eBioscience FoxP3/Transcription Factor Staining Buffer Set	Invitrogen	Cat#00-5523-00

**Experimental models: Cell lines**		

Lenti-X™ 293T Cell Line	TakaRa	Cat#632180
EL4	ATCC	Cat#TIB-39
2D2	This paper	N/A

**Experimental models: Organisms/strains**		

Mouse: C57BL/6-Tg(Tcra2D2,Tcrb2D2)1Kuch/J (B6)	The Jackson Laboratory	strain# 028276
Mouse: B6.Cg-Pdcd1tm1.1Shr/J (PD-1 knock out)	The Jackson Laboratory	strain# 028276
Mouse: C57BL/6-Tg(Tcra2D2,Tcrb2D2)1Kuch/J (2D2)	The Jackson Laboratory	strain# 006912

**Oligonucleotides**		

sgRNA sequences for targeting pdcd1 Forword: 5’- CACCG GACACACGGCGCAATGACAG -3’	This paper	N/A
sgRNA sequences for targeting pdcd1 Reverse: 5’- AAAC CTGTCATTGCGCCGTGTGTC C -3’	This paper	N/A
Primer for sequencing: 547-P1-F 5’-TTTCTGTTCTGCGCCGTTAC-3’	This paper	N/A
Primer for sequencing: 547-P2-F 5’-GATCAGCAACAGCGTGATG-3’	This paper	N/A
Primer for sequencing: 547-P3-R 5’-TTGCCTCTTCTGCGTTCG-3’	This paper	N/A
Primer for sequencing: 547-P4-F 5’-AGCCACCAAGGACACATACG-3’	This paper	N/A
Primer for sequencing: 547-P5-R 5’-GCCTTATTCCAAGCGGCTTC-3’	This paper	N/A

**Recombinant DNA**		

Plasmid: psPAX2	Addgene	Plasmid#12260
Plasmid: pHCMV-EcoEnv	Addgene	Plasmid#15802
Plasmid: pCDH-MSCV-PD1-FoxP3	This paper	N/A
Plasmid: pVSV-G	Addgene	Plasmid#138479
Plasmid: lentiCRISPRv2-mCherry	Addgene	Plasmid#99154

**Software and algorithms**		

Fiji	Fiji	http://www.figi.sc
Lenti-X GoStix App	Takara Bio	http://takarabio.com
GraphPad Prism 9	GraphPad	http://www.graphpad.com
Flowjo v10.10.0	BD Biosciences	http://www.flowjo.com/

### Experimental model and study participant details

#### Mice

Female, 8-10-week old PD-1^KO^ mice (B6.Cg-Pdcd1tm1.1Shr/J, strain # 028276), 2D2 TCR (TCRMOG) transgenic mice (C57BL/6-Tg(Tcra2D2,Tcrb2D2)1Kuch/J, strain # 006912), B6 mice (C57BL/6J, strain # 000664) were purchased from The Jackson Laboratory. Animal studies were conducted following a protocol approved by the Institutional Animal Care and Use Committee (IACUC) at the University of Utah. The protocol number of the research project approval document is 00001511. Upon arrival, mice were group-housed (five per cage) for at least one week prior to experimentation.

#### Cell lines

Lenti-X 293T cell line was purchased from Takara. 2D2 hybridoma cells are derived from 2D2 transgenic mice. EL4 cell line was purchased from ATCC, EL4 PD-1^KO^ cells were generated from EL4 cells.^38^ 2D2 PD-1^KO^ cells were generated from 2D2 cells, with detailed information provided in the method details. All these cell lines were maintained in DMEM medium supplemented with 10% of FBS. No authentication nor mycoplasma testing of the cell line was performed.

#### Mouse primary cells

Splenic and lymph node CD4^+^ T cells were isolated from female PD-1^KO^ mice, B6, or 2D2 mice using magnetic separation with the Mouse CD4^+^ T Cell Isolation Kit (Miltenyi Biotec) through negative selection and cultured in media of complete RPMI medium with 1% L-glutamine, 10% FBS and 100 U/mL hIL-2. CD4^+^ T cells from female PD-1^KO^ mice were used as parent cells for generating primary ^PD-1^CAR Tregs. CD4^+^ T cells from female B6, or 2D2 mice were used as stimulating cells for activation of ^PD-1^CAR Tregs or as target cells for ^PD-1^CAR Tregs. Female donors were selected to align with our planned in-vivo autoimmune disease studies (e.g., EAE), in which females typically show higher susceptibility and more consistent disease under our induction conditions. To stimulate the primary CD4^+^ T cells to express PD-1, the freshly isolated CD4^+^ T cells were activated on anti-CD3/CD28-coated plates (3 μg/mL) for three days to induce PD-1 expression. The stimulator cells were irradiated with X-ray (33Gy) using irradiator RadSource 2000 to inhibit further growth before stimulation assay.

### Method details

#### Generation of PD-1 ^KO^ 2D2 cell line via CRISPR/cas9 system

sgRNA sequences targeting mouse *Pdcd1* gene were cloned into the lentiCRISPRv2-mCherry plasmid (Addgene). This plasmid, along with psPAX2 (Addgene) and pVSV-G (Addgene), was co-transfected into Lenti-X cells for lentiviral packaging using the calcium phosphate precipitation method.^67^^,^^68^ Supernatants from the transfected Lenti-X cell cultures were collected 48 hours post-transfection and filtered through a 0.45 μm filter. The lentiviral particles were concentrated 10-20-fold using a Centricon with a 100 kDa cutoff (Millepore) by centrifugation at 1,000 g at 4°C for 15 minutes. For lentiviral transduction, 2D2 cells were plated at a density of 0.5 × 10^5^ cells/mL in a well of a 12-well plate. Spinoculation were performed in DMEM containing 10% FBS, 1% L-glutamine, and 8 μg/mL polybrene at 800*g* and 32°C for 30 minutes.^69^ After 5 days, cells were collected, mCherry-positive cells were sorted by FACSAria flow cytometry (BD Biosciences) and passaged to establish the PD-1^KO^ 2D2 cell line.

#### Design and construction of ^PD**-**1^CAR

The CAR antigen-binding domain (scFv) was derived from the 1A12 clone of anti-mouse PD-1, which exhibits high affinity for mouse PD-1 (*K*_d_ = 0.3 nM).^70^ The ^PD-1^CAR-Treg construct was designed by linking the scFv of anti-mouse PD-1 in-frame to the CD8 hinge and transmembrane domains, the 4-1BB (CD137) co-stimulatory domain, the CD28 co-activation domain, and the CD3 zeta signaling domain. A flexible linker of (GGGGS)3 was added between V_H_ and V_L_ sequences of the scFv to ensure proper folding and functionality. The construct also included a leader sequence from mouse CD8 to facilitate surface expression. Following the CAR sequence, a mouse FoxP3 coding sequence was added, separated by a P2A cleavage site. The entire CAR construct was codon-optimized (GeneArt) and cloned into the pCDH lentiviral plasmid backbone (Addgene) under the control of a mouse MSCV promoter. Additionally, an EF1α-driven GFP cassette was included in the construct as a selection marker.

#### Generation of ^PD**-**1^CAR Tregs vial lentiviral transduction

The pCDH lentiviral plasmid carrying the ^PD-1^CAR, along with psPAX2 (Addgene) and pHCMV-EcoEnv (Addgene), was co-transfected into Lenti-X cells to produce ecotropic envelope-pseudotyped lentivirus using the calcium phosphate precipitation method.^67^^,^^68^ Supernatants from the transfected Lenti-X cell cultures were collected at both 48 and 72 hours post-transfection and filtered through a 0.45 μm filter. Lentiviral particles were then concentrated around 20-fold using a Centricon with a 100 kDa cutoff (Millipore) by centrifugation at 1,000 g and 4°C. The viral titer was measured using Lenti-X GoStix Plus, following the manufacturer's instructions.

For generation of cell line-derived ^PD-1^CAR Treg by lentivirus transduction, 1 mL of 0.5 × 10^5^ 2D2 PD-1^KO^ cells was plated into a well of a 12-well plate and spinoculated with concentrated virus (MOI = 30) in DMEM supplemented with 10% FBS, 1% L-glutamine, and 8 μg/mL polybrene. Spinoculation was performed at 800 g and 32°C for 60 minutes. Four days after transduction, mCherry and GFP double-positive cells were sorted by flow cytometry and passaged to establish the ^PD-1^CAR cell line for characterization.

For generation of primary ^PD-1^CAR Treg by lentivirus transduction, CD4^+^ T cells isolated from PD-1^KO^ mouse were initially plated in a 12-well plate coated with 3 μg/mL of anti-CD3/CD28 and cultured for three days to stimulate activation. Subsequently, 1 mL of 5 × 10^5^ cells was transferred to a well of 6-well plate and spinoculated with the virus (MOI = 30) in the complete RPMI medium supplemented with 8 μg/mL polybrene at 800 g and 32°C for 90 minutes. A second spinoculation with the same conditions was performed the following day. Cells were then split on the next day with medium supplemented with 1000U hIL-2 and 50 ng/mL rapamycin. Five days after the initial transduction, GFP-positive cells were sorted by flow cytometry and cultured for an additional three days before conducting functional assays.

#### Cell-molecule binding assay

^PD-1^CAR Tregs and control cells were incubated with His-tagged recombinant PD-1 (R&D Systems) at varying concentrations (0.2, 1, 5 μg/mL) for 30 minutes at 4°C. After incubation, cells were washed and stained with APC-anti-His tag antibody (BioLegend) and analyzed by flow cytometry. The mean fluorescence intensity (MFI) of ^PD-1^CAR Tregs and control cells was quantified and analyzed to assess binding affinity.

#### Cell-cell conjugation assay

Engagement of ^PD-1^CAR Tregs with the target cells were assessed using immune cell conjugation assays, as previously described.^71^^,^^72^ Briefly, ^PD-1^CAR Tregs were labeled with 1 μM CFSE, and EL4 (PD-1^+^) or EL4 PD-1^KO^ (PD-1^-^) cells were labeled with 1 μM eFluor 670 in PBS at 1 × 10^6^ cells/mL, followed by incubation at 37°C for 15 min in the dark. Excess dye was quenched by adding 5× volume of DMEM with 5% FBS, incubating for 5 min, and washing twice with the medium. Labeled cells were resuspended in DMEM with 5% FBS at 1 × 10^6^ cells/mL, and 100 μL of ^PD-1^CAR Tregs was mixed with 100 μL of EL4 or EL4 PD-1^KO^ cells in a 3 mL flow cytometry tube. Cells were briefly centrifuged at 90g for 3 min and incubated at 37°C for 30 min to allow conjugate formation. The reaction was fixed by adding 300 μL of 4% PFA, followed by 10 min incubation at 37°C and centrifugation at 300 g for 5 min. Cells were washed with 2 mL of PBS, gently mixed, centrifuged again, and resuspended in 300 μL of PBS before flow cytometry analysis. Conjugated cells were identified as CFSE^+^ eFluor 670^+^ double-positive events, and the interaction was quantified as the percentage of the double positive cell doublets among total target cells (EL4 or EL4 PD-1^KO^).

#### Flow cytometric characterization of CAR Tregs

Surface molecule characterization of CAR Tregs or control cells was performed by staining cells with fluorescently labeled antibodies in PBS supplemented with 2% (w/v) FBS for 30 minutes at 4°C, followed by DAPI staining to exclude dead cells. For intracellular staining of proteins such as Ki67 and FoxP3, cells were first stained with Zombie Green or Zombie Violet fixable viability dyes (Biolegend), followed by surface antibody staining. Intracellular staining was then performed using the eBioscience FoxP3/Transcription Factor Staining Buffer Set (Thermo Fisher) according to the manufacturer’s protocol. Intracellular IFN-γ expression was assessed following treatment as indicated. On the day of analysis, cells were collected and incubated with Brefeldin A (5 μg/mL, BioLegend) for 5 hours to block protein secretion. As a positive control, CD4^+^ cells were treated with PMA (80 nM) and ionomycin (1 μg/mL, Stemcell Technologies) for 1 hour prior to the addition of Brefeldin A. Cells were then fixed and permeabilized using Fixation Buffer and 10× Permeabilization Wash Buffer (BioLegend) according to the manufacturer’s protocol, followed by staining for intracellular IFN-γ. Stained samples were acquired on a BD FACSCanto flow cytometer, and data were analyzed using FlowJo software (version 10, BD Biosciences).

#### Cytokine quantification

Supernatants were collected from co-cultures of ^PD-1^CAR Tregs or control cells with PD-1^+^ target cells at a 1:1 ratio after three days of incubation. Additionally, supernatants from ^PD-1^CAR Tregs stimulated with soluble PD-1 or plate-bound anti-CD3/CD28 were collected. All supernatants were stored at -80°C until further analysis. The concentrations of IL-10, TGFβ1, and CCN3 in the supernatants were quantified using ELISA kits (R&D Systems) following the manufacturer’s protocols. The concentrations of IFN-γ and IL-2 were quantified using LEGENDplex™ Multi-Analyte Flow Assay Kit (Biolegend) following the manufacturer’s protocols.

#### Proliferation suppression assay

For conventional proliferation inhibition assay, mosue CD4^+^ T cells were labeled with 1μM of CellTrace Far-Red (Thermo Fisher Scientific). Anti-CD3/CD28 beads (at a 1:10 cell-to-bead ratio) were then added, and the cells were cocultured with ^PD-1^CAR Tregs or control cells at specified ratios for four days. For PD-1^+^ cell proliferation inhibition assay, mouse CD4^+^ T cells were first activated on anti-CD3/CD28-coated plates (3 μg/mL) for three days to induce PD-1 expression. The preactivated cells were labeled with CellTrace Far-Red and cocultured with ^PD-1^CAR Tregs or control cells at specified ratios for four days. Conventional induced polyclone Treg cells were generated by culturing primary CD4^+^ T cells in the presence of anti-CD3/CD28 (3 μg/mL), hIL-2 (1000 U/mL), and hTGF-β (5 ng/mL) for five days. Proliferation was assessed by flow cytometry based on dye dilution. PE-anti-mouse PD-1 (clone 29F1A12) was used to stain PD-1^+^ cells. Cell proliferation was quantified using the Division Index (DI), calculated as the total number of cell divisions divided by the number of cells at the start of the culture. Proliferation inhibition was determined using the following equation:

Inhibition(%)=[(DIwithoutCARTregs−DIwithCARTregs)/DIwithoutCARTregs]×100%

#### OPC isolation and culture

OPCs were isolated from the cortices of neonatal B6 mice (postnatal day 7–9) using the Neural Tissue Dissociation Kit (P) (Miltenyi Biotec) and CD140a (PDGFRα) MicroBeads Kit (Miltenyi Biotec) with optimized protocols based on the manufacturer’s instructions. Briefly, cortices from three brains were dissected and combined, and the cerebellum, olfactory bulbs, and meninges were removed. The tissues were minced into small pieces and enzymatically digested using Enzyme P followed by Enzyme A. The resulting single-cell suspension was filtered through a 70 μm mesh. Cells were incubated with FcR blocker and CD140a (PDGFRα) MicroBeads before magnetic separation to isolate OPCs. The isolated OPCs were plated onto chamber slides coated with 50 μg/mL Poly-D-lysine at an appropriate density and cultured in OPC medium. The OPC medium consisted of MACS Neuro Medium supplemented with 2% MACS NeuroBrew®-21, 0.5% penicillin/streptomycin, 0.5 mM L-glutamine, 10 ng/mL PDGF-AA, and 10 ng/mL hFGF-2. Cultures were maintained by replacing 50% of the medium every other day for 7 days to support proliferation. On day 8, PDGF-AA and hFGF-2 were removed, and 1/5 of the medium was replaced with ^PD-1^CAR Treg-conditioned or control cell-conditioned media. The cells were cultured for an additional 3 days. Finally, cells were fixed with 4% PFA and stained with specific antibodies for immunofluorescence confocal microscopy analysis.

#### Immunofluorescence confocal microscopy

Immunoreactivity of OPC cells was assessed as previously described.^73^^,^^74^ Primary antibodies against goat-anti-human/mouse/rat Olig 2 (R&D Systems), rabbit anti-CNPase (Thermo Fisher Scientific), mouse anti-human/mouse/rat MBP (R&D Systems) were used at 8 μg/mL. Secondary fluorochrome antibodies were used at dilutions of 1:200, 1:400, and 1:300 for donkey anti-rabbit FITC-conjugated IgG, donkey Cy5-conjugated anti-mouse/rat IgG and donkey Cy3-conjugated anti-goat IgG, respectively (Jackson ImmunoResearch Laboratories). Slides with culture were mounted using VECTASHIELD anti-fade mounting medium (Vecto Laboratoties, Inc.). Fluorescent confocal images were acquired using a Zeiss LSM 710 microscope. Images were processed and analyzed using ImageJ to evaluate OPC differentiation and marker expression (Olig2, CNPase, MBP).

### Quantification and statistical analysis

Data are presented as mean ± SEM. Statistical analysis and graphing were performed using GraphPad Prism 9. The number of independent experiments, n values (representing individual sample) and statistical measurement can be found in the corresponding figure legends of each graph. Differences between groups were evaluated using a one-way ANOVA with post-hoc Tukey’s test or an unpaired two-tailed Student’s t-test for parametric data. A p-value of <0.05 was considered statistically significant.
