# Supplementary material for: 3-in-one PD-1CAR Tregs: A bioengineered cellular therapy for target engagement, activation, and immunosuppression with reparative potential
Source: iScience. 2025 Oct 3;28(11):113677. doi: 10.1016/j.isci.2025.113677 (PMC12556222; doi:10.1016/j.isci.2025.113677)
Supplement: Document S1. Figures S1–S5 [file mmc1.pdf]

iScience, Volume *28*

Supplemental information

3-in-one $^{PD-1}$CAR Tregs: A bioengineered cellular therapy for target engagement, activation, and immunosuppression with reparative potential

Shuyun Dong, Tianxiao Zhang, Yujia Zhai, Lauren C. Naatz, Noel G. Carlson, John W. Rose, Brian Evavold, and Mingnan Chen

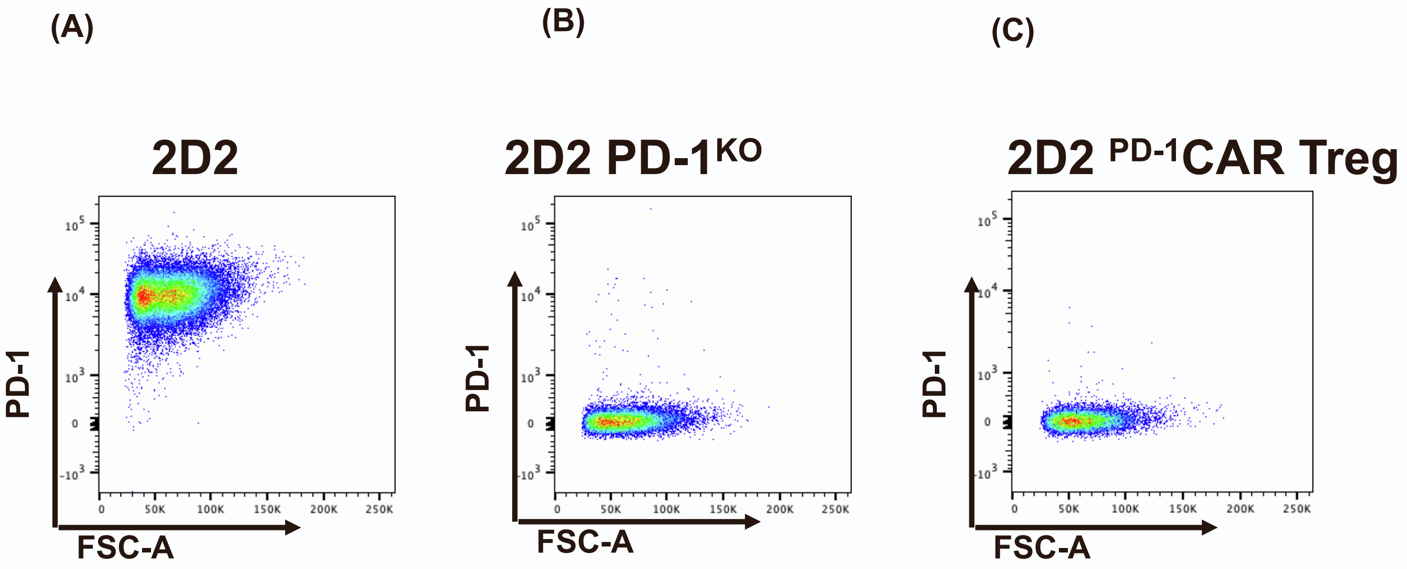

Figure S1. PD-1^{KO} is stable and consistent over time in 2D2 $^{PD-1}$CAR Tregs, related to Figure 1. (A-C) Flow cytometry dot plots showing PD-1 expression in wild-type 2D2 cells (A) , PD-1^{KO} 2D2 cells (B), and $^{PD-1}$CAR Tregs passaged for 10 generations (C), confirming the successful and consistent knockout of PD-1 in $^{PD-1}$CAR Tregs.

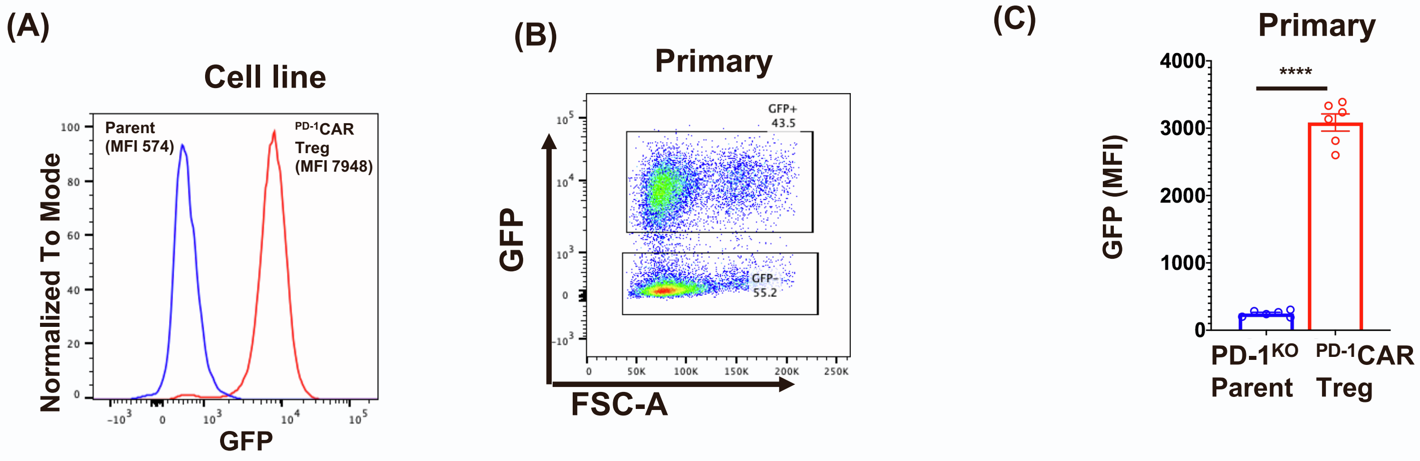

Figure S2. GFP expression was used as CAR sorting after $^{PD\text{-}1}$CAR virus transduction, related to Figure 1. (A) Representative flow cytometry histograms showing GFP expression in $^{PD\text{-}1}$CAR Tregs derived from PD-1^{KO} 2D2 cells, compared to untransduced parent cells. (B) Representative flow cytometry dot plots showing GFP expression in $^{PD\text{-}1}$CAR virus-transduced $CD4^+$ cells of PD-1^{KO} mice 7 days after transduction. (C) Bar graphs summarizing the MFI of GFP on $^{PD\text{-}1}$CAR Tregs derived from primary $CD4^+$ cells of PD-1^{KO} mice, compared to untransduced parent cells. n=6, **** *P* < 0.0001, as determined by Student's two-tailed t test.

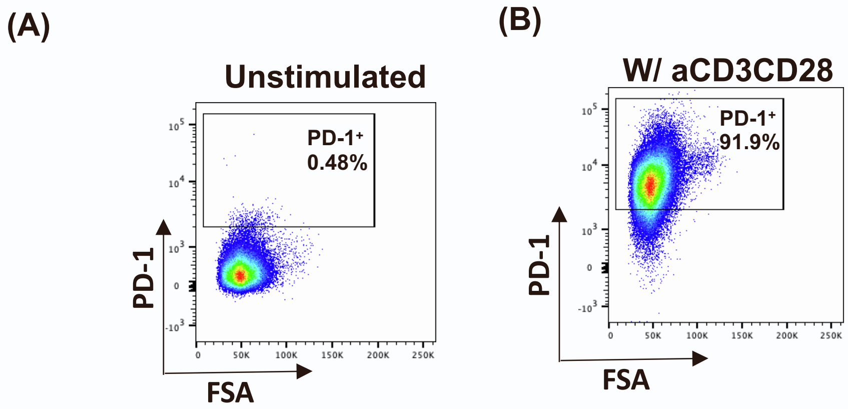

Figure S3. Generation of primary PD-1^{+} target cells by anti-CD3/CD28 stimulation, related to Figure 3. (A–B) Representative flow cytometry dot plots showing PD-1 expression on CD4^{+} T cells without stimulation (A) or after 3-day stimulation with plate-bound anti-CD3/CD28 (B).

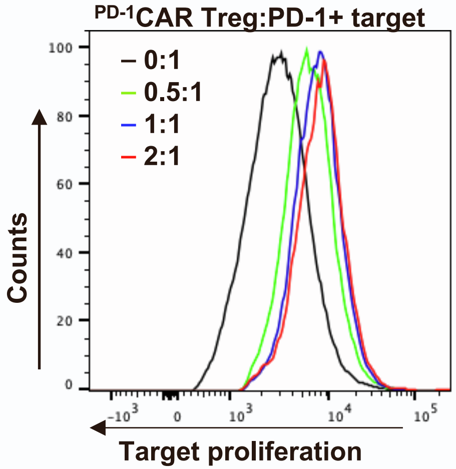

Figure S4. Primary $^{PD\text{-}1}$CAR Tregs inhibit proliferation of PD-1^{+} target cells, related to Figure 4. Representative flow cytometry histograms showing the proliferation of freshly isolated CD4^{+} T cells from 2D2 mice, pre-activated with plate-bound anti-CD3/CD28 for 3 days and labeled with CellTrace Far Red, after 4 days of co-culture with primary $^{PD\text{-}1}$CAR Tregs at varying ratios.

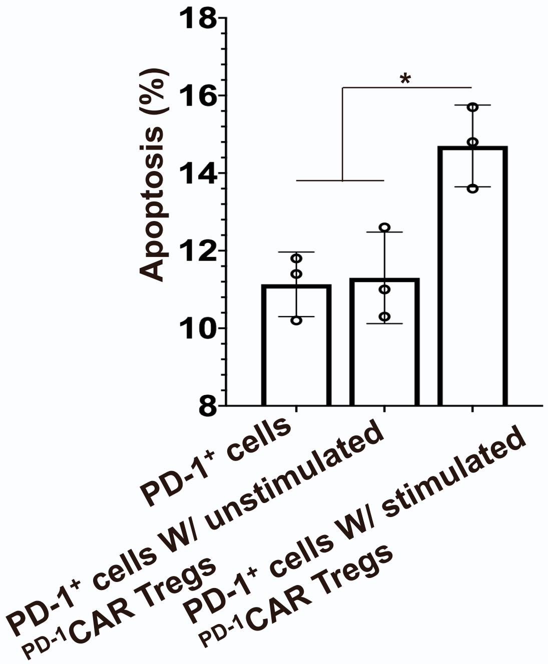

Figure S5. Apoptosis of PD-1$^+$ target cells in co-Culture with $^{PD-1}$CAR Tregs, related to Figure 4. Bar graphs showing the percentage of Annexin V$^+$ DAPI^{low} PD-1$^+$ cells (apoptotic population) after overnight co-culture with either stimulated or unstimulated $^{PD-1}$CAR Tregs at a 1:1 ratio. * $P < 0.05$, as determined by Student's two-tailed *t* test.
